# Cost Evaluation of Dried Blood Spot Home Sampling as Compared to Conventional Sampling for Therapeutic Drug Monitoring in Children

**DOI:** 10.1371/journal.pone.0167433

**Published:** 2016-12-12

**Authors:** Lisa C. Martial, Rob E. Aarnoutse, Michiel F. Schreuder, Stefanie S. Henriet, Roger J. M. Brüggemann, Manuela A. Joore

**Affiliations:** 1 Radboud university medical center, Department of Pharmacy, Nijmegen, The Netherlands; 2 Radboud Institute for Health Sciences, Nijmegen, The Netherlands; 3 Radboudumc Amalia Children’s Hospital, Radboud university medical center, Department of Pediatric Nephrology, Nijmegen, The Netherlands; 4 Radboudumc Amalia Children’s Hospital, Radboud university medical center, Department of Pediatric Infectious Diseases and Immunology, Nijmegen, The Netherlands; 5 Maastricht University Medical Center, Department of Clinical Epidemiology and Medical Technology Assessment (KEMTA), Maastricht, The Netherlands; Erasmus Universiteit Rotterdam, NETHERLANDS

## Abstract

Dried blood spot (DBS) sampling for the purpose of therapeutic drug monitoring can be an attractive alternative for conventional blood sampling, especially in children. This study aimed to compare all costs involved in conventional sampling versus DBS home sampling in two pediatric populations: renal transplant patients and hemato-oncology patients. Total costs were computed from a societal perspective by adding up healthcare cost, patient related costs and costs related to loss of productivity of the caregiver. Switching to DBS home sampling was associated with a cost reduction of 43% for hemato-oncology patients (€277 to €158) and 61% for nephrology patients (€259 to €102) from a societal perspective (total costs) per blood draw. From a healthcare perspective, costs reduced with 7% for hemato-oncology patients and with 21% for nephrology patients. Total savings depend on the number of hospital visits that can be avoided by using home sampling instead of conventional sampling.

## Introduction

Therapeutic drug monitoring (TDM) is an important way to individualize drug dosing for a variety of drug classes, such as anti-epileptics, antimicrobial agents (e.g. aminoglycosides and glycopeptides, azole antifungal agents), antimetabolites (e.g. methotrexate) and immunosuppressants (e.g. cyclosporine, tacrolimus) [1–3]. The purpose of concentration guided dose adaptation is to maintain drug exposure within predefined targets to ensure adequate efficacy and to decrease the likelihood of adverse events [4]. Conventionally, drug concentrations are measured in plasma, serum or whole blood obtained by venous sampling in a specialized healthcare facility [2, 3]. For patients treated on an outpatient basis, regular blood sampling can be challenging. It is time consuming, as patients have to attend their hospital outpatient clinic for blood sampling, and especially for children, frequent blood draws are associated with patient burden. For example, after renal transplantation, we perform TDM of immunosuppressants weekly in the first month post-discharge based on local practice, as guidelines on the frequency of TDM are lacking [5]. This frequency is slowly tapered to once every three months but this tapering usually takes 6–9 months.

Dried blood spot (DBS) sampling could be a more convenient and less costly alternative to conventional blood sampling. DBS sampling was first introduced for the screening of phenylketonuria in newborns (‘heel prick’ screening) in 1963 [6]. Especially over the last ten years, the development of DBS assays for drug concentration measurements has increased considerably [7, 8].

DBS sampling is used in different settings, from preclinical and clinical research including large epidemiological studies to clinical settings [9–15]. The advantages of DBS sampling over conventional venous sampling include the minimal invasive nature, the small amount of blood required, the stability of the sample and the ease of self-sampling at home. Moreover, DBS sampling allows for optimal sampling times (usual trough concentrations), which is often difficult to implement with outpatient visits. In addition, DBS sampling might potentially be cost-saving as no healthcare professionals are involved in the sampling process. Although this financial benefit of DBS sampling has been proposed in different clinical fields [16–18], no thorough cost evaluation on DBS home sampling for TDM has been performed so far.

The objectives of this study were (1) to develop an analytical framework for an integral cost evaluation of both conventional sampling and DBS home sampling; (2) to estimate and compare all costs associated with the two sampling methods and; (3) to identify factors that influence total cost. The results are presented in a format suitable for input into further health economic evaluations including cost-effectiveness analysis.

## Methods

### Setting

This cost evaluation is part of the PROTECT project funded by the Dutch Government, Rational Pharmacotherapy program, grant 836021012 from The Netherlands Organisation for Health Research and Development (ZonMW). PROTECT is coordinated by the Department of Pharmacy of the Radboud university medical center in close collaborations with the Departments of Pediatric Nephrology and Pediatric Infectious Diseases and Immunology of the Radboudumc Amalia Children’s Hospital, the Princess Máxima Center for oncology in Utrecht, the Wilhelmina Children’s Hospital in Utrecht and the Department of Pharmacy of the Maastricht University Medical Center.

#### Cases

Two pediatric patient populations, ‘cases’, were identified that are likely to benefit from DBS home sampling: children treated with immunosuppressants (tacrolimus, mycophenolic acid, cyclosporine) for the prevention of graft rejection after renal transplantation and children with (risk of) invasive fungal infections treated with azole antifungal agents (voriconazole, posaconazole, itraconazole). The main difference between these populations, relevant for this cost-evaluation, is the travel time from home to the hospital. Only three hospitals perform renal transplantation in children in The Netherlands for which their travel distance is expected to be higher than for pediatric hemato-oncology patients.

#### Framework for cost calculation

The literature was searched for articles to identify relevant cost items to be incorporated in the framework for the cost evaluation (the search can be found in S1 Text) and revealed no articles on the costs of DBS home sampling relevant for our cases.

As a result the framework was designed without a prior format by evaluating the whole process of blood sampling and TDM in patients treated on an outpatient basis. This included the subsequent steps: the request of the analysis, travelling to the healthcare facility, the process of blood sampling, analysis of the sample in the laboratory, interpretation of the results by the pharmacist, and feed-back to the patient. DBS home sampling contained an additional step of ‘instruction of DBS sampling’ as patients performing DBS home sampling require an introduction with instruction from a specialized healthcare professional (e.g. a nurse).

Input from patient-organizations representing the specific patient populations, pediatricians, hospital pharmacists, nurses, a hospital manager and a consultant hospital from “Consultancy Group Process Improvement and Innovation”from the Radboud university center was obtained to develop the framework for the two patient cases. Experts were asked to list all resources used, from a societal perspective, so irrespective of who incurred the costs. The time horizon was one single blood sample. This resulted in a list of resources used in the process of conventional sampling and a list of resources used in the process of DBS home sampling.

Resources used in each of the steps of the TDM process were subdivided into three categories, i.e. healthcare costs, patient costs and resources related to loss of productivity. Each category consisted of different items representing invested time, material, or overhead. The final framework was approved by the consulted experts. The sources of estimates for volume and cost units and basic assumptions on the base-case can be found in S1 Table.

### Analyses

#### Calculation sheet

The list of resources for both blood sampling methods was incorporated in a calculation sheet in Excel^™^ (Microsoft, USA). The calculation sheet contained all cost units, the volume per unit, and the cost per unit.

#### Base-case analysis

Total costs for a single blood sample for the purpose of TDM were computed by calculating all costs per cost unit (multiplying volume [e.g., minutes or parking tickets] and cost per unit); and summing up over all categories of healthcare costs, patient costs and resources related to loss of productivity; for both types of blood sampling: conventional sampling and DBS home sampling; and for the two patient cases. Given the learning effect in interpretation of the results after repeated analysis within the same patient, both patient cases were considered as new patients in the base-case. The step ‘instruction of DBS home sampling’ was not included in the integral costs of the DBS home sampling but was reported separately. The number of occasions of DBS home sampling required to earn back the costs of the instruction was also computed.

#### Sensitivity analyses

The following items were included in the sensitivity analysis: (1) patient travel time; (2) travelling by public transport instead of by car; (3) time spend in the hospital; (4) caregiver time valued as informal care; (5) caregiver time valued as loss of productivity for paid work; (6) sampling time; (7) costs of the laboratory analysis equals the formal Dutch national tariff (College Tarieven Gezondheidszorg, CTG tariff) [19]; (8) time related to review of the outcome of the sample analysis by the hospital pharmacist; (9) doctor’s time related to feedback to the patient. Resources (1–4) were unique to conventional sampling while resource (5) was unique to DBS home sampling. For details on the estimates used in the sensitivity analyses see S2 Table.

#### Scenario analyses

Total costs for blood sampling for the purpose of TDM during disease episode was calculated for each case for conventional sampling as well as for DBS sampling.

In pediatric nephrology, total costs on blood sampling for TDM of tacrolimus during the first three months post discharge after pediatric transplantation was calculated. This time period represents the most intense period in terms of outpatient visits with focus on blood analysis. Total costs for both a stable and an instable patient was computed. It was assumed that only instable patients may benefit from DBS homes sampling, as they require more intensive blood sampling. Details on the frequency of sampling can be found in S3 Table. Only cost associated with visits with the sole purpose of TDM were taken into account. In case visits were combined with other blood draws or outpatient visits, general costs such as nurse’s time or costs related to loss of productivity were not taken into account (best-case scenario). In case additional sampling was required, such as in instable patients, all costs were taken into account. For conventional sampling, these additional blood samples were assumed to be drawn in a shared care center, assumed to be closer to a patient’s home [20].

In hemato-oncology, a treatment episode of invasive pulmonary aspergillosis (six months) with voriconazole was used to calculate all costs related to blood sampling for the purpose of TDM. Six months was considered representative based on literature [21] and our own experience in treating such infections in this population. A learning effect was taken into account for interpretation of the result of the voriconazole concentration by the hospital pharmacist from the third sample onwards, resulting in less time spent on interpretation and counseling (10 vs 20 minutes). This learning effect reflects the current situation in our hospital: interpretation and counseling on azole antifungal TDM is performed by a selected number of hospital pharmacists who know recurring patients. As hemato-oncology patients require regular blood sampling for determination of biochemical parameters (liver function, electrolytes), only costs associated with sampling for the purpose of voriconazole TDM were taken into account (best-case scenario). In case extra sampling was required, such as in the first month of therapy for dose individualization, all costs as included in the base-case were taken into account. Details on the design of these two additional scenarios including frequency and location of sampling are presented in S3 Table.

## Results

### Base case analysis

For children with renal transplants treated with immunosuppressants, total societal costs for conventional sampling amount €259 for one sample while home sampling costs are €102 for a sample (Tables 1 and 2). Total savings per sample are depicted in the last column, i.e. for a nephrology patient, DBS home sampling results in €23 saving of patient costs, €107 saving of costs related to loss of productivity and €27 saving of healthcare costs. Clearly, the difference is mainly driven by a reduction in costs related to loss of productivity of the parent accompanying his/her child to the hospital for the sampling process. The total societal costs of conventional sampling in children treated with azole antifungal agents amount €277 while home sampling amounts €158. Again, the difference between both methods is mainly associated with a decrease in costs related to loss of productivity and less to patient costs and healthcare costs. The difference in costs related to DBS sampling between the two patient cases lies mainly in doctors/pharmacists time related to interpretation of the result and contacting the patient (Tables 1 and 2).

**Table 1:** Base-case analysis for population (1): pediatric nephrology patients.

Cost unit	Conventional sampling			DBS home sampling			Difference DBS—Conventional
	Volume/unit	Cost/unit (€)	Costs (€)	Volume/unit	Cost/unit (€)	Costs (€)	(€)
**Request of the analysis**							
*Healthcare costs*							
Doctor orders analysis	3.25 min	1.89	6.16	3.25 min	1.89	6.16	0
Overhead	44%		2.71	44%		2.71	0
**Subtotal request of analysis**			9			9	**0**
**Blood drawing**							
*Patient costs*							
Travel expenses car	104 km	0.19	19.88			NA	-19.88
Travel expenses parking	1 ticket	3.02	3.02			NA	-3.02
*Costs related to loss of productivity*							
Travel time	145 min	0.58	84.37			NA	-84.37
Time in hospital	45 min	0.58	26.22			NA	-26.22
Home sampling			NA	10 min	0.23	2.35	2.35
Send the sample by mail			NA	6 min	0.23	1.41	1.41
*Healthcare costs*							
Sampling by nurse	15 min	0.54	8.15			NA	-8.15
Overhead	44%		3.58			NA	-3.58
Sampling material	1 unit	6	6	1 unit	5.69	5.69	-0.31
**Subtotal blood drawing**			151			9	**-142**
**Laboratory**							
*Healthcare costs*							
Cost of laboratory analysis	1 unit	50.00	50.00	1 unit	50.00	50.00	0
Review by hospital pharmacist	5 min	1.89	9.47	3 min	1.89	5.68	-3.79
Overhead	44%		4.17	44%		2.50	-1.67
**Subtotal laboratory**			64			58	**-6**
**Feedback to patient**							
*Healthcare costs*							
Doctor processes result in medical record	7 min	1.89	13.26	3.5 min	1.89	6.63	-6.63
Patient contacted	6 min	1.89	11.37	6 min	1.89	11.37	0
Overhead	44%		10.84	44%		7.92	-2.92
**Subtotal feed-back to patient**			35			26	**-9**
**Total patient costs**			23			0	-23
**Total costs related to loss of productivity**			111			4	-107
**Total healthcare costs**			126			99	-27
**Total societal costs**			**259**			**102**	**-157**

Discrepancies between multiplications and sums may be due to rounding. DBS *dried blood spot*.

**Table 2:** Base-case analysis for population (2): pediatric hemato-oncology patients.

Cost unit	Conventional sampling			DBS home sampling			DifferenceDBS—Conventional
	Volume/unit	Cost/unit (€)	Costs (€)	Volume/unit	Cost/unit (€)	Costs (€)	(€)
**Request of the analysis**							
*Healthcare costs*							
Doctor orders analysis	3.25 min	1.89	6.16	3.25 min	1.89	6.16	0
Overhead	44%		2.71	44%		2.71	0
**Subtotal request**			9			9	0
**Blood drawing**							
*Patient costs*							
Travel expenses distance	78 km	0.19	14.91			NA	-14.91
Travel expenses parking	1 ticket	3.02	3.02			NA	-3.02
*Costs related to loss of productivity*							
Travel time	114 min	0.58	66.19			NA	-67.59
Time in hospital	45 min	0.58	26.22			NA	-26.22
Home sampling			NA	10 min	0.23	2.35	2.33
Send the sample by mail			NA	6 min	0.23	1.41	1.4
*Healthcare costs*							
Sampling by nurse	15 min	0.54	8.15			NA	-8.15
Overhead	44%		3.58			NA	-3.58
Sampling material	1 unit	6	6	1 unit	5.69	5.69	-0.31
**Subtotal blood drawing**			128			9	-119
**Laboratory**							
*Healthcare costs*							
Cost laboratory analysis	1 unit	50.00	50.00	1 unit	50.00	50.00	0
Review by hospital pharmacist	20 min	1.89	37.89	20 min	1.89	37.89	0
Overhead	44%		16.67	44%		16.67	0
**Subtotal laboratory**			105			105	**0**
**Feedback to patient**							
*Healthcare costs*							
Doctor processes result in medical record	7 min	1.89	13.26	7 min	1.89	13.26	0
Patient contacted	6 min	1.89	11.37	6 min	1.89	11.37	0
Overhead	44%		10.84	44%		10.84	0
**Subtotal feedback to patient**			35			35	**0**
**Total patient costs**			18			0	-18
**Total costs related to loss of productivity**			92			4	-88
**Total healthcare costs**			167			155	-12
**Total societal costs**			**277**			**158**	**-119**

Discrepancies between multiplications and sums may be due to rounding. DBS *dried blood spot*.

**Fig 1:**
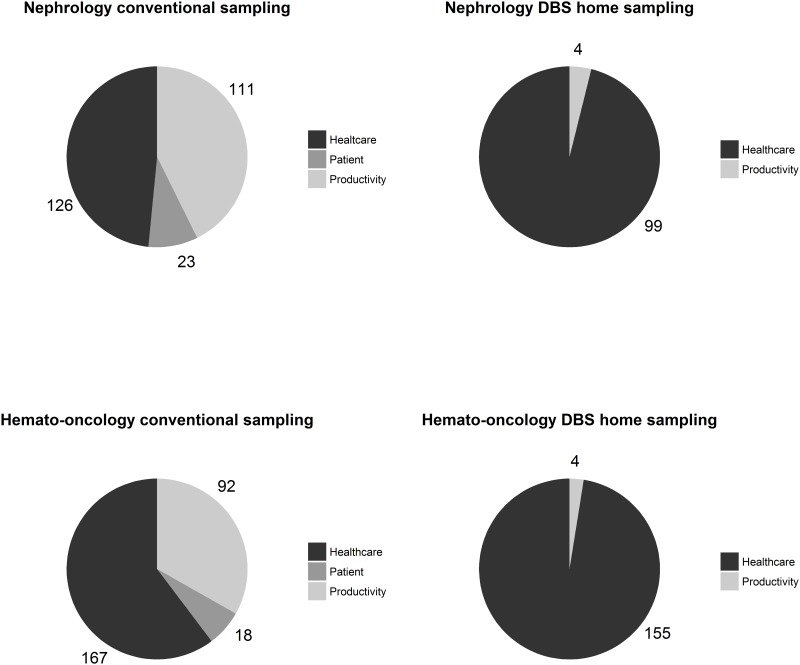
Pie chart of costs in euro’s (€) ordered by perspective. *DBS* dried blood spot.

Pie charts depicted in Fig 1 represent the distribution of patient costs, costs related to loss of productivity and healthcare costs with conventional sampling and DBS home sampling for both cases. While for conventional blood sampling healthcare costs represent 49–60% of total costs, this is >97% with DBS home sampling. With home sampling, patient costs are absent and costs related to loss of productivity are reduced with >95% as compared to conventional sampling.

The instruction of the patient and his/her parent by a specialized nurse is estimated to be €163 for children with renal transplants and €139 for children treated with azole antifungal agents, as shown in Table 3. The variation in costs is driven by differences in travel time. The investment of the instruction of home sampling by the specialized nurse is almost completely earned back at the second occasion of home sampling, both from a societal as from a healthcare perspective.

**Table 3:** Resources on instruction of home sampling for both populations.

	Nephrology			Hemato-oncology			Difference between populations Nephrology—Hemato-oncology
Instruction of DBS finger prick	Volume (unit)	Cost/unit (€)	Costs (€)	Volume (unit)	Cost/unit (€)	Costs (€)	(€)
*Patient costs*							
Travel expenses car	104 km	0.19	19.88	78 km	0.19	14.91	4.97
Travel expenses parking	1 ticket	3.02	3.02	1 ticket	3.02	3.02	0
subtotal			**23**			**18**	**5**
*Costs related to loss of productivity*							
Travel time	145 min	0.58	84.37	114 min	0.58	66.19	18.18
Time in hospital	45 min	0.58	26.22	45 min	0.58	26.22	0
subtotal			**111**			**92**	**19**
*Healthcare costs*							
Time of nurse for instruction	30 min	0.54	16.29	30 min	0.54	16.29	0
Overhead	44%		7.17	44%		7.17	0
Instruction material	1 unit	5.69	5.69	1 unit	5.69	5.69	0
subtotal			**29**			**29**	**0**
**Total**			**163**			**139**	**24**

Discrepancies between multiplications and sums may be due to rounding. DBS *dried blood spot*.

### Sensitivity analysis

**Fig 2:**
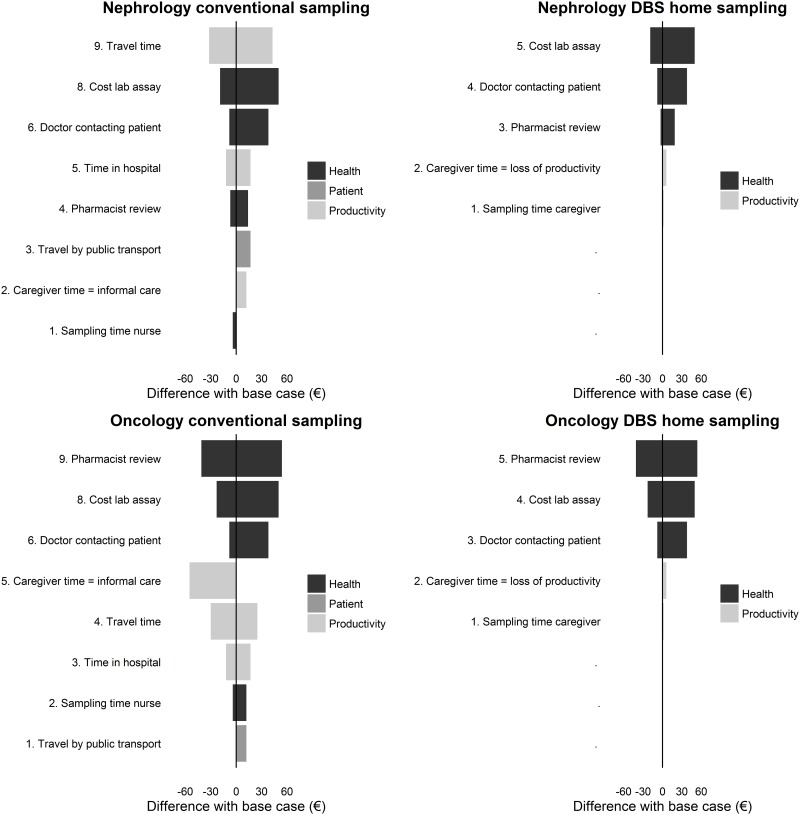
One-way sensitivity analysis of the impact of the variation of different items on total societal cost as compared to the base-case. DBS *dried blood spot*.

Tables 4 and 5 and tornado plots in Fig 2 show how total costs vary with pessimistic or opportunistic inputs. The main factor of influence is the time of the hospital pharmacist and doctor related with review of the outcome of the sample analysis and feedback to the patient, respectively. For example, if the time of feedback to the patient increased from 6 minutes (base case) to 20 minutes, total costs of conventional blood drawing increased with 15% from €259 to €297 for children with immunosuppressant therapy. The costs of sample analysis in the laboratory are also influential. If the prize doubled to €100 total costs increased with 18–19% for conventional sampling and with about 32–49% for DBS home sampling. The time for a nurse to take a sample showed to be less influential on total costs, doubling the sampling time from 15 to 30 minutes led to about 4–5% increase in total costs.

**Table 4:** Sensitivity analysis population (1): pediatric nephrology patients.

Conventional sampling	Costs of the item (€)	Total cost (€)	Difference with base case (€)	DBS home sampling	Costs of the item (€)	Total cost (€)	Difference with base case (€)
*Resources regarding loss of productivity*							
**Travel time patient**							
Base case	84	259	NA				NA
Optimistic scenario 1st quartile	52	227	-32				NA
Pessimistic scenario 3rd quartile	128	303	43				NA
**Productivity loss time in hospital**							
Base case (45 min)	26	259	NA				NA
Optimistic scenario (25 min)	15	248	-12				NA
Pessimistic scenario (75 min)	44	277	17				NA
**Caregiver time is valued as informal care**				**Caregiver time is valued as loss of paid work**			
Base case: valued as loss of paid work	111	259	NA	Base case: valued as informal care	4	102	NA
Caregiver time valued as informal care	45	193	-66	Caregiver time valued as loss of paid work	9	108	6
*Patient costs*							
**Patient travels by public transport**							
Base case: car	23	259	NA				NA
Public transport	40	276	17				NA
							NA
*Healthcare costs*							
**Sampling time nurse**				**Sampling time caregiver**			
Base case (15 min)	12	259	NA	Base case (sampling takes 10 min)	2	102	NA
Optimistic scenario (sample takes 10 min)	8	255	-4	Optimistic scenario (5 min)	1	101	-1
Pessimistic scenario (sample takes 30 min)	23	271	12	Pessimistic scenario (20 min)	5	105	2
**Costs of the lab analysis**				**Costs of the lab analysis**			
Base case	50	259	NA	Base case	50	102	NA
CTG tariff	31	241	-19	CTG tariff	31	84	-19
Twice base case	100	309	50	Twice base case	100	152	50
**Costs related to review by pharmacist**				**Costs related to review by pharmacist**			
Base case (5 min)	14	259	NA	Base case (3 min)	8	102	NA
Optimistic scenario (2.5 min)	7	252	-7	Optimistic scenario (2 min)	5	100	-3
Pessimistic scenario (10 min)	27	273	14	Pessimistic scenario (10 min)	27	121	19
**Time related to contacting patient**				**Time related to contacting patient**			
Base case (total 6 min)	16	259	NA	Base case (total 6 min)	16	102	NA
Optimistic scenario (total 3 min)	8	251	-8	Optimistic scenario (total 3 min)	8	94	-8
Pessimistic scenario (20 min)	54	297	38	Pessimistic scenario (20 min)	54	141	38

Discrepancies between multiplications and sums may be due to rounding. DBS *dried blood spot*.

**Table 5:** Sensitivity analysis population (2): pediatric oncology patients.

Conventional sampling	Costs of the item (€)	Total cost (€)	Difference with base case (€)	DBS home sampling	Costs of the item (€)	Total cost (€)	Difference with base case (€)
*Resources regarding loss of productivity*							
**Travel time patient**							
Base case	66	277	NA				NA
Optimistic scenario 1st quartile	36	247	-30				NA
Pessimistic scenario 3rd quartile	91	302	25				NA
**Productivity loss time in hospital**							
Base case (45 min)	26	277	NA				NA
Optimistic scenario (25 min)	15	265	-12				NA
Pessimistic scenario (75 min)	44	294	17				NA
*Patient costs*							
**Patient travels by public transport**							
Base case: car	18	277	NA				NA
Public transport	30	289	12				NA
							NA
**Caregiver time is valued as informal care**				**Caregiver time is valued as loss of paid work**			
Base case: valued as loss of paid work	92	277	NA	Base case: valued as informal care	4	158	NA
Caregiver time valued as informal care	37	222	-55	Caregiver time valued as loss of paid work	9	164	6
*Healthcare costs*							
**Sampling time nurse**				**Sampling time caregiver**			
Base case (15 min)	12	277	NA	Base case	2	158	NA
Optimistic scenario (sample takes 10 min)	8	273	-4	Optimistic scenario (5 min)	1	157	-1
Pessimistic scenario (sample takes 30 min)	23	289	12	Pessimistic scenario (20 min)	5	161	2
**Costs of the lab analysis**				**Costs of the lab analysis**			
Base case	50	277	NA	Base case	50	158	NA
CTG tariff	27	254	-23	CTG tariff	27	135	-23
Twice base case	100	327	50	Twice base case	100	208	50
**Costs related to review by pharmacist**				**Costs related to review by pharmacist**			
Base case (20 min)	55	277	NA	Base case	55	158	NA
Optimistic scenario (5 min)	14	236	-41	Optimistic scenario (5 min)	14	117	-41
Pessimistic scenario (40 min)	109	331	54	Pessimistic scenario (40 min)	109	213	54
**Time related to contacting patient**				**Time related to contacting patient**			
Base case (6 min)	16	277	NA	Base case (6 min)	16	158	NA
Optimistic scenario (3 min)	8	269	-8	Optimistic scenario (total 3 min)	8	150	-8
Pessimistic scenario (20 min)	54	315	38	Pessimistic scenario (20 min)	54	196	38

Discrepancies between multiplications and sums may be due to rounding. DBS *dried blood spot*.

Valuing all caregiver time as informal care in the situation of conventional sampling in a healthcare facility, decreased total costs with 20–25%. When all caregiver time was valued as loss of productivity instead of informal care in the situation of DBS home sampling, this was associated with a 4–6% increase in total cost.

### Scenario analyses

Total costs associated with blood sampling for the purpose of TDM of tacrolimus in the pediatric nephrology patient in the first three months post discharge after renal transplantation amount to €756 for a stable patient conventionally sampled. This is based on a total of 7 samples (see S3 Table). As all samples for tacrolimus TDM are supposed to be drawn together with routine sample taking, DBS home sampling does not play a role in regular patient care and hence does not impact costs in a stable transplant patient. For the instable transplant patient, a total of 11 samples will be drawn which results in a total cost of €1401 for blood sampling for the purpose of TDM. DBS home sampling can reduce this total cost to €1226 in case three samples are taken at home. These three samples were based on consultation with two pediatric nephrologists.

Total costs for TDM of voriconazole in a hemato-oncology patient amount to €3972 based on a total of 28 samples taken conventionally during a disease episode of 6 months. DBS home sampling can reduce total costs to €3553 in case four samples are taken at home.

## Discussion

This is the first study evaluating the costs of conventional blood sampling in a healthcare facility and comparing this to DBS home sampling for the purpose of TDM. Costs for a single blood sample as well as costs for a complete disease episode were estimated in two populations. From a societal perspective, total costs for a single blood draw with DBS home sampling were substantially lower compared to conventional blood sampling in a healthcare facility, i.e., 2.5 fold lower for nephrology patients and 1.8 fold lower for hemato-oncology patient. Total costs associated with conventional sampling for the purpose of TDM in the first three or six months post transplantation and in hemato-oncology patients with an invasive fungal infection respectively, could be reduced 1.1 fold.

Factors influencing total costs per blood draw were the time of the pharmacist and physician, the costs of sample analysis, and the valuation of caregiver time (informal care of lost productivity from paid labour). The scenario analyses per disease episode showed that the saving as a result of DBS largely depends on the proportion of hospital visits that can be avoided.

Cost reductions are dependent on how many outpatient visits can be avoided, as children may travel to the hospital not only for drug concentration measurement but also for sampling of biochemical parameters and/or for an outpatient visit with their physician. This largely depends on the population and the clinical status of individual patient within this population. Implementation of DBS home sampling requires alertness and flexibility of the treating physician and the healthcare system in order to decide when best to apply home sampling and when not. Especially upon titration of the drug, home sampling can substitute outpatient visits. Currently, hemato-oncology patients treated from an outpatient basis have weekly appointments with their hemato-oncologist. For each additional sample required for monitoring of blood concentrations, patients require to travel to our center for blood sampling as local general hospitals have no facilities in assessment of azole blood concentrations. Clearly, DBS home sampling can reduce costs for every additional sample. Those savings are mainly true from a societal point-of-view as they mostly cover savings in loss of productivity and loss of patient (travel) costs.

As it stands, no thorough cost evaluation regarding DBS home sampling for TDM has been published. DBS sampling was only hypothesized to be cost-saving as compared to conventional sampling [17, 18]. One study evaluated the costs of different sampling methods for the assessment of prenatal alcohol exposure and concluded that DBS sampling from the newborn was cheaper as compared to conventional venous sampling of the mother or meconium analysis. DBS sampling was cost-saving in terms of personnel involved in venous sampling and shipping costs when compared to venous sampling of the mother [16]. DBS sampling resulted in a reduction in nurse’s time of 11 minutes, comparable to our 15 minutes time reduction. Our results confirm that DBS home sampling is cost-saving.

The calculated costs and figures are inevitably associated with uncertainty. Sensitivity analyses identified several items that specifically influenced total costs. The costs of the laboratory analysis of the sample depend on several local factors including equipment, cost prices of the laboratory, number of samples per run and number of runs per year. It is assumed that no specific analytical equipment is required for the analysis of DBS samples as mostly liquid chromatography mass-spectrometry is used for the analysis of both plasma and DBS samples[8]. For the base-case scenario a general price of €50 was used. The sensitivity analyses showed that total costs decreased substantially by using a national maximal tariff (CTG tariff) [19] that applies for the analysis of outsourced samples in The Netherlands. Inversely, doubling the price to €100 also impacted total costs considerably. National tariffs do not always cover all costs involved with measurement of the sample, especially for drugs that are infrequently measured such as azole antifungal agents. A €100 fee probably better reflects the real costs involved in sample measurement. The current analysis did not take into account investments required for developing a DBS method, which may be more costly than plasma method development. On the other hand, several approaches have been made to speed-up the analytical method such as automated flow-through desorption or reduced sample pre-treatment resulting in a cost-effective analytical method[22, 23]. These technical innovations will further reduce analysis costs in the future.

Another item especially influencing total cost was the time spend by the physician and hospital pharmacist for the interpretation of the result of the analysis and feed-back to the patient respectively, which is explained by the relatively high salary costs of these healthcare professionals. Although this finding has no clinical consequences and is equally true for both conventional sampling as DBS home sampling, it can be a good starting point for strategies to further decrease costs associated with blood sampling (e.g. efficient administration processes, good clinical handover).

As home sampling has not been implemented yet, assumptions were made on the time required for parents to sample their child. Total costs were only marginally influenced by time spent by the parent on sampling, as shown by the sensitivity analysis. Of note, these cost were indeed influenced by how this caregiver time was valued, i.e. as loss of productivity for paid work or as informal care. The majority of total cost involved in DBS home sampling included healthcare costs.

Implementing a DBS home sampling method will on one hand lead to cost savings from a societal and patient perspective, as patients have less travel costs and reduced loss of productivity. Societal costs will decline and social health security may benefit from reduced healthcare costs. It may on the other hand lead to a loss of income for a healthcare facility. It is difficult to predict how this latter aspect will impact hospital related costs. This will become more clear when DBS sampling becomes more common practice. Our analysis and results are valid for the social and economical situation in the Netherlands. Probably similar trends can be expected upon extrapolation to other countries with similar health-care systems but this is only hypothetical.

Adequate sampling times are very important for good interpretation of the exposure to immunosuppressants and azoles: preferably trough concentrations (before a next dose of the drug) are measured [24]. With conventional sampling during an outpatient visit it is difficult to ‘capture’ this time point as patients usually take their medication in the (early) morning before the outpatient visit. With home sampling this trough concentration can be ‘captured’ facilitating the interpretation by the treating physician and hospital pharmacist. DBS home sampling is expected to result in better samples in terms of sampling moments, to increase the interpretability of the outcome and to facilitate subsequent decision making on dose adaptations. Other advantages of DBS sampling are not easily represented in costs, such as the minimal volume required or the less invasive nature of sampling.

DBS home sampling may reduce costs even more when biochemical parameters such as renal function parameters for nephrology patients or liver function tests in case of treatment with azole antifungal agents are measured along with drug concentrations. Those parameters are important to take into account in further studies. At this moment, most biochemical parameters require a plasma or serum sample to be taken at the hospital. DBS assays for creatinine have been published but some are associated with issues of precision [25, 26]. Combining home sampling for the purpose of TDM with assessment of disease specific parameters will probably result in increased benefit for the patient as more outpatient visits may be avoided. This will most likely also lead to lower societal costs. Clearly, DBS home sampling has a great potential for implementation in regular patient care and this can be further improved by combining measurement of different components in one sample. When implementing a DBS home sampling method for quantitative analysis of compounds, careful instruction with regular feed-back on the spot quality and thorough screening of the spot before processing the sample in the laboratory is important. Improperly collected samples may impact quality of care as the analytical outcome may be modified resulting in improper clinical decision. Moreover, improperly collected samples may negatively impact costs.

We would like to stress that DBS sampling is a nice alternative in case patients travel to the hospital only for blood sampling and do not have an appointment with their physician or (specialized) nurse practitioner. It is obvious that home sampling should not replace doctor’s appointments.

Based on the current cost evaluation, DBS home sampling can be considered cost-saving. Whether DBS home sampling is more efficient, depends on the accuracy and precision of the DBS assay compared to the conventional (plasma) assay [27–30]. Alongside our clinical study on efficacy of DBS sampling, a cost-effectiveness of DBS home sampling is performed. Also patient preferences and the experience regarding DBS home sampling are subject of study. We anticipate that children would prefer a finger prick over regular sampling and that parents would be ready to perform finger prick sampling of their child. Literature shows that DBS home sampling in a clinical study among female adult cancer patients was successful with about 70% participation [18]. Another study on islet autoantibody screening in patients aged 12–38 years showed that patients would prefer DBS home sampling if this would avoid visits to the clinic, despite that DBS finger prick sampling was more painful [12].

## Conclusion

This study is the first evaluation comparing the costs involved in regular blood sampling with the costs involved in DBS home sampling for the purpose of TDM. From societal perspective, total costs per blood draw decrease drastically with home sampling. Patient costs are reduced to zero and cost related to loss of productivity are decreased with >95%. Scenario analyses revealed that total cost savings depends heavily on how many visits can be avoided. Based on the results of this study we can conclude that DBS home sampling is associated with a reduction in costs both from a healthcare as from a societal perspective, but the size of the reduction differs considerably per individual patient in a population.

## Supporting Information

S1 Raw data Regular blood draw. (PDF)

S2 Raw data Sensitivity analysis nephrology. (PDF)

S3 Raw data Sensitivity analysis oncology. (PDF)

S4 Raw data Disease episode nephrology. (PDF)

S5 Raw data Disease episode oncology. (PDF)

S1 Table Sources of volumes and cost items. (DOCX)

S2 Table Ranges used in sensitivity analyses. (DOCX)

S3 Table Details of scenario analyses. (DOCX)

S1 Text Literature search, sources of estimates for volume unit and cost units, basic assumptions of the cost analysis. (DOCX)
