# Supplementary material for: Cost Evaluation of Dried Blood Spot Home Sampling as Compared to Conventional Sampling for Therapeutic Drug Monitoring in Children
Source: PLoS One. 2016 Dec 12;11(12):e0167433. doi: 10.1371/journal.pone.0167433 (PMC5152813; doi:10.1371/journal.pone.0167433)
Supplement: S1 Raw data — (PDF) [file pone.0167433.s001.pdf]

## REGULAR BLOOD DRAWING

| Cost unit | Volume | Unit | Price | Costs | Within healthc? | P/M | Source | Source |
|---|---|---|---|---|---|---|---|---|
| *parts of the process of regular blood drawing* | | | */volume* | | yes/no | | volume | price |
| **Method development plasma/whole blood UPLC/MS** | | | | | | | | |
| *not included* | | | | | | | | |
| **Request of the analysis** | | | | | | | | |
| Doctor orders the analyis in the EPIC | 3.25 | min | 1.89 | 6.16 | yes | p | Dr. Michiel | Handleiding |
| Overhead | 0.44 | % | 6.16 | 2.71 | no | o | NA | Handleiding |
| **subtotal request of analysis** | | | | 9 | | | | |
| **Blood drawing** | | | | | | | | |
| Loss of productivity: travel time car NEFROLOGY | 145 | min | 0.58 | 84.37 | no | | Schretlen S | Handleiding |
| Loss of productivity: travel time car ONCOLOGY | 114 | min | 0.58 | 66.19 | no | | Schretlen S | Handleiding |
| Loss of productivity: time in the hospital | 45 | min | 0.58 | 26.22 | no | | patient org | Handleiding |
| Pt&parent travel expenses car NEFROLOGY | 104 | km | 0.19 | 19.88 | no | | Schretlen S | Handleiding |
| Pt&parent travel expenses parking | 1 | ticket | 3.02 | 3.02 | no | | na | Handleiding |
| Pt&parent travel expenses car ONCOLOGY | 78 | km | 0.19 | 14.91 | no | | Schretlen k | Handleiding |
| Nurse takes venous sample | 15 | min | 0.54 | 8.15 | yes | p | Marjo vd D | Navraag bij I |
| Overhead personnel | 0.44 | % | 8.15 | 3.58 | yes | o | NA | Handleiding |
| Material | 1 | unit | 6.04 | 6.04 | yes | m | Marjo vd D | Input Marjo |
| **subtotal blood drawing nefrology** | | | | 151 | | | | |
| **subtotal blood drawing oncology** | | | | 128 | | | | |
| **Laboratory** | | | | | | | | |
| Costs analysis per sample | 1 | euro | 50.00 | 50.00 | yes | m | NA | Hosptial pha |
| Pharmacist reviews results, communication to prescriber NEFROLOGY | 5 | min | 1.89 | 9.47 | yes | p | interviews | Handleiding |
| Pharmacist reviews results, communication to prescriber ONCOLOGY | 20 | min | 1.89 | 37.89 | | p | interviews | Handleiding |
| Overhead NEFROLOGY | 0.44 | % | 9.47 | 4.17 | | o | NA | Handleiding |
| Overhead ONCOLOGY | 0.44 | % | 37.89 | 16.67 | | o | NA | Handleiding |
| **subtotal laboratory nefrology** | | | | 64 | | | | |
| **subtotal laboratory oncology** | | | | 105 | | | | |
| **Feed-back to patient** | | | | | | | | |
| Doctor gets the result and processes this information in EPD | 7 | min | 1.89 | 13.26 | yes | p | Dr. Michiel | Handleiding |
| Pt&parent are contacted by doctor: result and dose adaptation if necessary | 6 | min | 1.89 | 11.37 | yes | p | Dr. Michiel | Handleiding voor koste |
| Overhead personnel | 0.44 | % | 24.63 | 10.84 | | o | NA | Handleiding voor koste |
| **subtotal feed-back** | | | | 35 | | | | |
| **subtotal productivity** | | | | 111 | | | | |
| **subtotal patient** | | | | 23 | | | | |
| **subtotal health** | | | | 126 | | | | |
| **total NEFROLOGY** | | | | 259 | | | | |
| **subtotal productivity** | | | | 92 | | | | |
| **subtotal patient** | | | | 18 | | | | |
| **subtotal health** | | | | 167 | | | | |
| **total ONCOLOGY** | | | | 277 | | | | |
| Repeat from 'Request of the analysis' | | | | | | | | |

## DRIED BLOOD SPOT BLOOD DRAWING

| Cost unit | Volume | Unit | Price | Costs | Within healthc? | P/M | Source | Source |
|---|---|---|---|---|---|---|---|---|
| *parts of the process of DBS blood drawing* | | | */volume eenheid* | | yes/no | | volume | prijs |
| **Method development DBS** | | | | | | | | |
| *not included* | | | | | | | | |
| **Instruction of DBS fingerprick** | | | | | | | | |
| Loss of productivity: travel time NEFROLOGY | 145 | min | 0.58 | 84.37 | no | | Schretlen 6 | Handleidin |
| Loss of productivity: travel time ONCOLOGY | 114 | min | 0.58 | 66.19 | no | | Schretlen k | Handleidin |
| Loss of productivity: time in the hospital | 45 | min | 0.58 | 26.22 | no | | patient org | Handleidin |
| Pt&parent travel expenses public transport NEFROLOGY | 208 | km | 0.19 | 39.76 | no | | Schretlen 6 | Handleidin |
| Pt&parent travel expenses car NEFROLOGY | 104 | km | 0.19 | 19.88 | no | | Schretlen 6 | Handleidin |
| Pt&parent travel expenses parking | 1 | ticket | 3.02 | 3.02 | no | | NA | Handleidin |
| Pt&parent travel expenses public transport ONCOLOGY | 156 | km | 0.19 | 29.82 | no | | Schretlen k | Handleidin |
| Pt&parent travel expenses car ONCOLOGY | 78 | km | 0.19 | 14.91 | no | | Schretlen k | Handleidin |
| Nurse gives instruction | 30 | min | 0.54 | 16.29 | yes | p | estimation | Navraag bi |
| Overhead personnel | 0.44 | % | 16.29 | 7.17 | yes | o | NA | Handleidin |
| Material | 1 | unit | 5.69 | 5.69 | yes | p | DBSL | DBSL |
| **subtotal instruction DBS nefrology** | | | | 163 | | | | |
| **subtotal instruction DBS oncology** | | | | 139 | | | | |
| **Request of the analysis** | | | | | | | | |
| Doctor orders the analyis in the EPIC, 'open order' without specified date | 3.25 | min | 1.89 | 6.16 | yes | p | | Handleidin |
| Overhead | 0.44 | % | 6.16 | 2.71 | | o | | Handleidin |
| **subtotal request of analysis** | | | | 9 | | | | |
| **Blood drawing by means of finger prick** | | | | | | | | |
| Loss of productivity/ 'mantelzorg' finger prick (patient together with parent) | 10 | min | 0.23 | 2.35 | no | | estimation | Handleidin |
| Material for finger prick 1 kit (filter paper, pricker, gauze, plaster, envelope) | 1 | pcs | 5.69 | 5.69 | yes | m | DBSL | DBSL |
| Time spend sending the sample with envelope by regular mail (TNT) | 6 | min | 0.23 | 1.41 | no | | TNT | http://www |
| **subtotal blood drawing** | | | | 9 | | | | |
| **Laboratory** | | | | | | | | |
| Costs analysis per sample NEFROLOGY | 1 | euro | 50.00 | 50.00 | yes | m | NA | Hosptial pl |
| Pharmacist reviews results, communication to prescriber NEFROLOGY | 3 | min | 1.89 | 5.68 | yes | p | interviews | Handleidin |
| Pharmacist reviews results, communication to prescriber ONCOLOGY | 20 | min | 1.89 | 37.89 | | p | interviews | Handleidin |
| Overhead NEFROLOGY | 0.44 | % | 5.68 | 2.50 | | o | NA | Handleidin |
| Overhead ONCOLOGY | 0.44 | % | 37.89 | 16.67 | | o | NA | Handleidin |
| **subtotal laboratory nefrology** | | | | 58 | | | | |
| **subtotal laboratory oncology** | | | | 105 | | | | |
| **Feed-back to patient** | | | | | | | | |
| Doctor gets the result and processes this information in EPD NEFRO | 3.5 | min | 1.89 | 6.63 | yes | p | Dr. Michiel | Handleidin |
| Doctor gets the result and processes this information in EPD ONCOLOGY | 7 | min | 1.89 | 13.26 | | | | Handleidin |
| Pt&parent are contacted by doctor: result and dose adaptation if necessary | 6 | min | 1.89 | 11.37 | yes | p | Dr. Michiel | Handleidin |
| Overhead personnel NEPHROLOGY | 0.44 | % | 18.00 | 7.92 | | o | NA | Handleidin |
| Overhead personnel ONCOLOGY | 0.44 | % | 24.63 | 10.84 | | o | NA | Handleiding voor kostenonderzoek. 8 sept2015. chapter 3.6 |
| **subtotal feed-back nefro** | | | | 26 | | | | |
| **subtotal feedback onco** | | | | 35 | | | | |
| **subtotal productivity** | | | | 4 | | | | |
| **subtotal patient** | | | | 0 | | | | |
| **subtotal health** | | | | 99 | | | | |
| **total NEFROLOGY** | | | | 102 | | | | |
| **subtotal productivity** | | | | 4 | | | | |
| **subtotal patient** | | | | 0 | | | | |
| **subtotal health** | | | | 155 | | | | |
| **total ONCOLOGY** | | | | 158 | | | | |
| Repeat from 'Request of the analysis' | | | | | | | | |
