# Supplementary material for: Cost Evaluation of Dried Blood Spot Home Sampling as Compared to Conventional Sampling for Therapeutic Drug Monitoring in Children
Source: PLoS One. 2016 Dec 12;11(12):e0167433. doi: 10.1371/journal.pone.0167433 (PMC5152813; doi:10.1371/journal.pone.0167433)
Supplement: S2 Raw data — (PDF) [file pone.0167433.s002.pdf]

Sensitivity analyses

Conventional sampling nefrology

Cost unit		Costs (€)	Total cost
**Productivity travel time patient**			
	Base case	84	**259**
	Optimistic scenario 1st quartile	52	227
	Pessimistic scenario 3rd quartile	128	303
**Patient costs: travels by public transport**			
	Base case: car	23	259
	Public transport	40	276
**Productivity loss time in hospital**			
	Base case	26	259
	Optimistic scenario ready in 25 min	15	248
	Pessimistic scenario 1 hour waiting	44	277
**Sampling time nurse**			
	Base case (15 min)	12	259
	Optimistic scenario (sample takes 10 mins)	8	255
	Pessimistic scenario (sample takes 30 mins)	23	271
**Costs of the lab analysis**			
	Base case	50	259
	CTG tarief	31	241
	Twice base case	100	309
**Costs related to review by pharmacist**			
	Base case (5 min)	14	259
	Optimistic scenario (2.5 min)	7	252
	Pessimistic scenario (10 min)	27	273
**Time related to contacting patient**			
	Base case (total 6 min)	16	259
	Optimistic scenario (total 3 min)	8	251
	Pessimistic scenario (20 min)	54	297
	**Additional scenario's**		
**All patient time is 'mantelzorg' time**			
	Base case	111	259
	all time is 'mantelzorg'	45	193

All patient time is 'productivity loss' = base case

Base case	111	259
all time is 'productivity loss'	111	259

Sensitivity analyses

DBS home sampling nefrology

Difference with base case		Costs (€)	Total cost
NA			
-32			
43			
NA			
17			
NA			
-12			
17			
	**Sampling time parent**		
NA	Base case (10 min)	2	102
-4	Optimistic scenario (5 min)	1	101
12	Pessimistic scenario (20 min)	5	105
	**Costs of the lab analysis**		
NA	Base case	50	102
-19	CTG tarief	31	84
50	Twice base case	100	152
	**Costs related to review by pharmacist**		
NA	Base case (3 min)	8	102
-7	Optimistic scenario (2 min)	5	100
14	Pessimistic scenario (10 min)	27	121
	**Time related to contacting feed back to patient**		
NA	Base case (total 6 min)	16	102
-8	Optimistic scenario (total 3 min)	8	94
38	Pessimistic scenario (20 min)	54	141
	**Additional scenario's**		
	**All patient time is 'mantelzorg' time = base case**		
NA	Base case	4	102
-66	all time is 'mantelzorg'	4	102

	**All patient time is 'productivity loss'**			
NA		Base case	4	102
0		all time is 'productivity loss'	9	108

Difference with base case
NA
NA
NA
NA
NA
NA
NA
NA
NA
-1
2
NA
-19
50
NA
-3
19
NA
-8
38
NA
0

NA

6
