# Supplementary material for: Cost Evaluation of Dried Blood Spot Home Sampling as Compared to Conventional Sampling for Therapeutic Drug Monitoring in Children
Source: PLoS One. 2016 Dec 12;11(12):e0167433. doi: 10.1371/journal.pone.0167433 (PMC5152813; doi:10.1371/journal.pone.0167433)
Supplement: S3 Raw data — (PDF) [file pone.0167433.s003.pdf]

Sensitivity analyses

Conventional sampling oncology

Cost unit		Costs (€)
**Productivity travel time patient**		
	Base case	66
	Optimistic scenario 1st quartile	36
	Pessimistic scenario 3rd quartile	91
**Patient costs: travel by public transport**		
	Base case: car	18
	Public transport	30
**Productivity loss time in hospital**		
	Base case	26
	Optimistic scenario sample was taken in 10 mins, only 15 mins waiting time	15
	Pessimistic scenario 1 hour waiting	44
**Sampling time nurse**		
	Base case (15 min)	12
	Optimistic scenario (sample takes 10 mins)	8
	Pessimistic scenario (sample takes 30 mins)	23
**Costs of the lab analysis**		
	Base case	50
	CTG tarief	27
	Twice base case	100
**Costs related to review by pharmacist**		
	Base case (20 min)	55
	Optimistic scenario (5 min)	14
	Pessimistic scenario (40 min)	109
**Time related to contacting feed back to patient**		
	Base case (6 min)	16
	Optimistic scenario (total 3 min)	8
	Pessimistic scenario (20 min)	54
**All patient time is 'mantelzorg' time**		
	Base case	92
	all time is 'mantelzorg'	37
**All patient time is 'productivity loss'**		
	Base case	92
	all time is 'productivity loss'	92

Sensitivity analyses

		DBS home sampling oncology		
Total cost	Difference with base case		Costs (€)	Total cost
277	NA			
247	-30			
302	25			
277	NA			
289	12			
277	NA			
265	-12			
294	17			
		**Sampling time parent**		
277	NA	Base case (10 min)	2	158
273	-4	Optimistic scenario (5 min)	1	157
289	12	Pessimistic scenario (20 min)	5	161
		**Costs of the lab analysis**		
277	NA	Base case	50	158
254	-23	CTG tarief	27	135
327	50	Twice base case	100	208
		**Costs related to review by pharmacist**		
277	NA	Base case	55	158
236	-41	Optimistic scenario (5 min)	14	117
331	54	Pessimistic scenario (40 min)	109	213
		**Time related to contacting feed back to patient**		
277	NA	Base case (6 min)	16	158
269	-8	Optimistic scenario (total 3 min)	8	150
315	38	Pessimistic scenario (20 min)	54	196
		**All patient time is 'mantelzorg' time**		
277	NA	Base case	4	158
222	-55	all time is 'mantelzorg'	4	158
		**All patient time is 'productivity loss'**		
277	NA	Base case	4	158
277	0	all time is 'productivity loss'	9	164

Difference with base case
NA
NA
NA
NA
NA
NA
NA
NA
NA
-1
2
NA
-23
50
NA
-41
54
NA
-8
38
NA
0
NA
6

Schretlen k

Handleiding voor kostenonderzoek. 8 sept2015. hst 5.1.2. 0.19 ct per km.
