# Supplementary material for: Cost Evaluation of Dried Blood Spot Home Sampling as Compared to Conventional Sampling for Therapeutic Drug Monitoring in Children
Source: PLoS One. 2016 Dec 12;11(12):e0167433. doi: 10.1371/journal.pone.0167433 (PMC5152813; doi:10.1371/journal.pone.0167433)
Supplement: S4 Raw data — (PDF) [file pone.0167433.s004.pdf]

Disease episode nefrology, first 3 months

costs conventional sampling tacrolimus nephrology

stable patient

Cost unit	costs for one sampling moment	n=	cost for the whole period
	€	7	€
**Request of the analysis**	9		**62**
**Laboratory**	64		445
**Feed back to patient**	35		248
**total stable patient**	108		**756**

instable patient

Cost unit	costs for one sampling moment	n=	cost for the whole period
**sampling in Rumc**	€	8	€
**Request of the analysis**	9		**71**
**Laboratory**	64		509
**Feed back to patient**	35		284
total sampling Rumc	108		864
sampling in shared care center	€	3	€
**Request of the analysis**	9		27
**Sampling in other center**			
productivity loss patient	48		143
costs travelling	6		17
sampling time nurse	12		35
sampling material	6		18
**Total sampling**	71		213
**Laboratory**	64		191
**Feed back to patient**	35		106
total shared care sampling	179		537
**Total instable conventional**			**1401**

costs partially DBS home sampling nephrology

stable patient

Cost unit	costs for one sampling moment	n=	cost for the whole period
	€	0	€
**NA**			

instable patient

Cost unit	costs for one sampling moment	n=	cost for the whole period
**sampling in Rumc**	€	8	€
**Request of the analysis**	9		71
**Laboratory**	64		509
**Feed back to patient**	35		284
total sampling Rumc	108		864
**Instruction home sampling**		1	
**Parent time**	**26**		26
**Nurse time**	**23**		23
**Material**	**6**		6
total	**55**		55
**Home sampling**	€	3	€
**Request of the analysis**	**9**		**27**
**Sampling at home**			
productivity loss patient	4		11
sampling material	6		17
**Total sampling**	9		28
**Laboratory**	58		175
**Feed back to patient**	26		78
total home sampling	**102**		**307**
**total instable DBS**			**1226**
