# Supplementary material for: Cost Evaluation of Dried Blood Spot Home Sampling as Compared to Conventional Sampling for Therapeutic Drug Monitoring in Children
Source: PLoS One. 2016 Dec 12;11(12):e0167433. doi: 10.1371/journal.pone.0167433 (PMC5152813; doi:10.1371/journal.pone.0167433)
Supplement: S5 Raw data — (PDF) [file pone.0167433.s005.pdf]

Disease episode oncology 6 mo

costs conventi

stable patient

Cost unit

first sample

Request of the analysis
Laboratory
Feed back to patient
total

second sample [extra appointment]

Request of the analysis
Blood drawing
Laboratory
Feed back to patient
total

Sample no. 4,6,8 [extra appointment]

Request of the analysis
Blood drawing
Laboratory
sample
time pharmacist (10 min)
overhead
Total laboratory
Feed back to patient
total

Regular samples (no 3,5,7 and rest of 5 months)

Request of the analysis
Laboratory
sample
time pharmacist (10 min)
overhead
total laboratory

Feed back to patient

total

Total

onths

ional sampling voriconazole

costs for one sampling moment	n=	cost for the whole period
€	1	€
9		9
105		105
35		35
149		149
€	1	€
9		9
128		128
105		105
35		35
277		277
€	3	€
9		27
128		384
50		150
19		57
8		25
77		232
35		106
250		749
€	23	€
9		204
50		1150
19		436
8		192
77		1778

35			816
122			2797
		€	**3972**

costs partially DBS home sampling voriconazole

stable patient

Cost unit		costs for one sampling moment	n=
**first sample [hospital]**		€	1
**Request of the analysis**		9	
**Laboratory**		105	
**Feed back to patient**		35	
	total	149	
**Instruction home sampling**			1
**Parent time in hospital**		26	
**Nurse time**		23	
**Material**		6	
	total	55	
**second sample [home]**		€	1
**Request of the analysis**		9	
**Blood drawing at home**		9	
**Laboratory**		105	
**Feed back to patient**		35	
	total	158	
**Sample no. 4,6,8 [home]**		€	3
**Request of the analysis**		9	
**Blood drawing at home (productivity loss)**		9	
**Laboratory**			
	sample	50	
	time pharmacist (10 min)	19	
	overhead	8	
**total laboratory**		77	
**Feed back to patient**		35	
	total	131	
**Regular samples (no 3,5,7 and rest of 5 months)**		€	23
**Request of the analysis**		9	
**Laboratory**			
	sample	50	
	time pharmacist (10 min)	19	
	overhead	8	
**total laboratory**		77	

**Feed back to patient**		35	
	total	122	
	**Total**		€

cost for the whole period
€
9
105
35
149
26
23
6
55
€
9
9
105
35
158
€
27
28
150
57
25
232
106
393
€
204
1150
436
192
1778

816

2797

3553
