# Supplementary material for: Cost Evaluation of Dried Blood Spot Home Sampling as Compared to Conventional Sampling for Therapeutic Drug Monitoring in Children
Source: PLoS One. 2016 Dec 12;11(12):e0167433. doi: 10.1371/journal.pone.0167433 (PMC5152813; doi:10.1371/journal.pone.0167433)
Supplement: S1 Table — (DOCX) [file pone.0167433.s006.docx]

Supplementary Table 1a. Sources of volumes and cost items.

| **Cost unit** | **Source** | |
| --- | --- | --- |
|  | **Volume** | **Cost** |
| **Request of the analysis** |  |  |
| *Healthcare costs* |  |  |
| Doctor orders analysis | Interview with pediatricians (n=5) | Manual cost analysis [1] |
| Overhead | Manual cost analysis [1] | NA |
|  |  |  |
| **Conventional blood drawing** |  |  |
| *Patient costs* |  |  |
| Travel expenses car | Data Radboudumc, patient population 2015 | Manual cost analysis [1] |
| Travel expenses parking | 1 parking ticket | Manual cost analysis [1] |
| *Costs related to loss of productivity* |  |  |
| Travel time | Data Radboudumc | Manual cost analysis [1] |
| Time in hospital | Survey with patient organizations (n=4) | Manual cost analysis [1] |
| Home sampling | Estimation | Manual cost analysis [1] |
| Send the sample by mail | http://www.postnl.nl 500 m walking = 6 min walking | Manual cost analysis [1] |
| *Healthcare costs* |  |  |
| Sampling by nurse | Interview with nurse (n=2) and coordinator housing controller | Manual cost analysis [1] & CAO UMC 2013-2015 |
| Overhead | Manual cost analysis [1] |  |
| Sampling material conventional | Data Radboudumc from coordinator housing | Data Radboudumc from coordinator housing |
| Sampling material home sampling | Estimation | Data on file, from Dried Blood Spot Laboratory (DBSL) Geleen, The Netherlands |
|  |  |  |
| **Laboratory** |  |  |
| *Healthcare costs* |  |  |
| Cost of laboratory analysis | Estimation | Assumption of general price |
| Review by hospital pharmacist | Interview with hospital pharmacists (in training) (n=7) | Manual cost analysis [1] |
| Overhead | Manual cost analysis [1] |  |
|  |  |  |
| **Feedback to patient** |  |  |
| *Healthcare costs* |  |  |
| Doctor processes result in medical record | Interview with pediatricians (n=5) | Manual cost analysis [1] |
| Patient contacted | Interview with pediatricians (n=5) | Manual cost analysis [1] |
| Overhead | Manual cost analysis [1] | Manual cost analysis [1] |
|  |  |  |
| **Instruction of DBS finger prick** |  |  |
| *Patient costs* |  |  |
| Travel expenses car | Data Radboudumc, patient population 2015 | Manual cost analysis [1] |
| Travel expenses parking | 1 parking ticket | Manual cost analysis [1] |
| *Costs related to loss of productivity* |  |  |
| Travel time | Data Radboudumc | Manual cost analysis [1] |
| Time in hospital | Estimation | Manual cost analysis [1] |
| *Healthcare costs* |  |  |
| Time of nurse for instruction | Estimation |  |
| Overhead | Manual cost analysis [1] |  |
| Instruction material | Estimation | Data on file, from Dried Blood Spot Laboratory (DBSL), Geleen, The Netherlands |

Reference Manual cost analysis

1. Zorginstituut Nederland. Kostenhandleiding: Methodologie van kostenonderzoek en referentieprijzen voor economische evaluaties in de gezondheidszorg. 2015.
