# Supplementary material for: Cost Evaluation of Dried Blood Spot Home Sampling as Compared to Conventional Sampling for Therapeutic Drug Monitoring in Children
Source: PLoS One. 2016 Dec 12;11(12):e0167433. doi: 10.1371/journal.pone.0167433 (PMC5152813; doi:10.1371/journal.pone.0167433)
Supplement: S2 Table — (DOCX) [file pone.0167433.s007.docx]

Supplementary Table 2. Ranges used in sensitivity analyses.

| **Cost unit** | **Optimistic scenario** | **Base case** | **Pessimistic scenario** | **Source of volume** | **Source of cost** |
| --- | --- | --- | --- | --- | --- |
| Productivity: travel time patient | 1st quartile | Median travel time | 3rd quartile | Data Radboudumc | Manual cost analysis [1] |
| Patient costs: patient travels by public transport | NA | By car | Public transport | NA | Manual cost analysis [1] |
| Productivity loss time in hospital | 25 min | 45 min | 75 min | Interviews with patient organizations and nurses | Manual cost analysis [1] |
| Conventional sampling Productivity: caregiver time valued as informal care | Time valued as informal care | Time valued as loss of paid work | NA | NA | Manual cost analysis [1] |
| DBS home sampling:  Productivity: caregiver time valued as loss of paid work | NA | Time valued as informal care | Time valued as loss of paid work | NA | Manual cost analysis [1] |
| Healthcare: sampling time nurse | 10 min | 15 min | 30 min | Interviews with nurses (n=2) | Manual cost analysis [1] |
| Healthcare: costs of the lab analysis | CTG tariff | €50,- | €100,- | empirical | For CTG-tariff: [2] |
| Healthcare: costs related to review by pharmacist | Nephrology: 2.5 min  Hemato-oncology:  5 min | Nephrology:  5 min  Hemato-oncology:  20 min | Nephrology: 20 min  Hemato-oncology:  40 min | Interviews with hospital  pharmacists (in training) (n=7) | Manual cost analysis [1] |
| Healthcare: time related to contacting patient | 3 min | 6 min | 20 min | Interviews with pediatricians (n=5) | Manual cost analysis [1] |
| Productivity: home sampling time patient | 5 min | 10 min | 20 min | Estimation | Manual cost analysis [1] |

References:

1. Zorginstituut Nederland. Kostenhandleiding: Methodologie van kostenonderzoek en referentieprijzen voor economische evaluaties in de gezondheidszorg. 2015.

2. Nederlandse Zorg Autoriteit (NZA). Tarieventabel DBC-zorgproducten en overige producten - per 1 januari 2015.
