# Supplementary material for: Cost Evaluation of Dried Blood Spot Home Sampling as Compared to Conventional Sampling for Therapeutic Drug Monitoring in Children
Source: PLoS One. 2016 Dec 12;11(12):e0167433. doi: 10.1371/journal.pone.0167433 (PMC5152813; doi:10.1371/journal.pone.0167433)
Supplement: S3 Table — (DOCX) [file pone.0167433.s008.docx]

Supplementary Table 3. Details of scenario analyses.

Table 3a: Total costs related to sampling for TDM of tacrolimus the first 3 months post discharge after renal transplantation

| **Stable patient Nephrology** | | | | | | | | |
| --- | --- | --- | --- | --- | --- | --- | --- | --- |
|  | **Conventional sampling** | | | | **Home sampling** | | | |
| **Cost unit** | **cost one sample** | n= | **cost for the whole period** | | NA | | | |
| **Sampling in Rumc** | **€** | 7 | **€** | |  |  |  |  |
| Request of the analysis | 9 |  | 62 | |  |  |  |  |
| Laboratory | 64 |  | 445 | |  |  |  |  |
| Feed back to patient | 35 |  | 248 | |  |  |  |  |
|  |  |  |  | |  |  |  |  |
| **Total stable patient** | **108** |  | **756** | |  |  |  |  |
| **Instable patient Nephrology** | | | | | | | | |
|  | **Conventional sampling** | | | **Home sampling** | | | | |
| **Cost unit** | **cost one sample** | n= | **cost for the whole period** |  | | **cost one sample** | n= | **cost for the whole period** |
| **Sampling in Rumc** | **€** | 8 | **€** | **Sampling in Rumc** | | **€** | 8 | **€** |
| Request of the analysis | 9 |  | 71 | Request of the analysis | | 9 |  | 71 |
| Laboratory | 64 |  | 509 | Laboratory | | 64 |  | 509 |
| Feed back to patient | 35 |  | 284 | Feed back to patient | | 35 |  | 284 |
| **Total sampling Rumc** | 108 |  | 864 | **Total sampling Rumc** | | 108 |  | 864 |
|  |  |  |  |  | |  |  |  |
|  |  |  |  | **Instruction home sampling** | | **€** | 1 | **€** |
|  |  |  |  | Productivity loss | | 26 |  | 26 |
|  |  |  |  | Instruction nurse | | 23 |  | 23 |
|  |  |  |  | Material | | 6 |  | 6 |
|  |  |  |  | **Total instruction** | | 55 |  | 55 |
|  |  |  |  |  | |  |  |  |
| **Sampling in shared care center** | **€** | 3 | **€** | **Home sampling** | | **€** | 3 | **€** |
| Request of the analysis | 9 |  | 27 | Request of the analysis | | 9 |  | 27 |
| Sampling |  |  |  | Sampling at home | |  |  |  |
| productivity loss patient | 48 |  | 143 | productivity loss patient | | 4 |  | 11 |
| costs travelling | 6 |  | 17 | sampling material | | 6 |  | 17 |
| sampling time nurse | 12 |  | 35 |  | |  |  |  |
| sampling material | 6 |  | 18 |  | |  |  |  |
| Total sampling shared care | 71 |  | 213 | Total sampling at home | | 9 |  | 28 |
| Laboratory | 64 |  | 191 | Laboratory | | 58 |  | 175 |
| Feed back to patient | 35 |  | 106 | Feed back to patient | | 26 |  | 78 |
| **Total shared care sampling** | 179 |  | 537 | **Total home sampling** | | 102 |  | 307 |
|  |  |  |  |  | |  |  |  |
| **Total instable patient conventional** |  | | **1401** | **Total instable patient with home sampling** | |  | | **1226** |

Rumc: Radboud University Medical Center.
Discrepancies between multiplications and sums may be due to rounding.
Table 3b: Total costs related to sampling for TDM of voriconazol in hemato-oncology patient, treatment period of 6 months.

| **Conventional sampling stable patient** | | | | **DBS home sampling stable patient** | | | |
| --- | --- | --- | --- | --- | --- | --- | --- |
| Cost unit | **cost one sample** | **n=** | **cost for the whole period** |  | **cost one sample** | **n=** | **cost for the whole period** |
| **First sample** | € | 1 | € | **First sample** | € | 1 | € |
| Request of the analysis | 9 |  | 9 | Request of the analysis | 9 |  | 9 |
| Laboratory | 105 |  | 105 | Laboratory | 105 |  | 105 |
| Feed back to patient | 35 |  | 35 | Feed back to patient | 35 |  | 35 |
| **Total first sample** | 149 |  | 149 | **Total first sample** | 149 |  | 149 |
|  |  |  |  |  |  |  |  |
|  |  |  |  | **Instruction home sampling** | **€** | 1 | **€** |
|  |  |  |  | Productivity loss | 26 |  | 26 |
|  |  |  |  | Instruction nurse | 23 |  | 23 |
|  |  |  |  | Material | 6 |  | 6 |
|  |  |  |  | **Total instruction** | 55 |  | 55 |
|  |  |  |  |  |  |  |  |
| **Second sample [extra appointment]** | € | 1 | € | **Second sample [home]** | € | 1 | € |
| Request of the analysis | 9 |  | 9 | Request of the analysis | 9 |  | 9 |
| Blood drawing | 128 |  | 128 | Blood drawing at home | 9 |  | 9 |
| Laboratory | 105 |  | 105 | Laboratory | 105 |  | 105 |
| Feed back to patient | 35 |  | 35 | Feed back to patient | 35 |  | 35 |
| **Total second sample** | 277 |  | 277 | **total** | 158 |  | 158 |
|  |  |  |  |  |  |  |  |
| **Sample no. 4,6,8 [extra appointment]** | € | 3 | € |  | € | 3 | € |
| Request of the analysis | 9 |  | 27 | Request of the analysis | 9 |  | 27 |
| Blood drawing | 128 |  | 384 | Blood drawing at home | 9 |  | 28 |
| Laboratory |  |  |  | Laboratory |  |  |  |
| sample | 50 |  | 150 | sample | 50 |  | 150 |
| time pharmacist (10 min) | 19 |  | 57 | time pharmacist (10 min) | 19 |  | 57 |
| overhead | 8 |  | 25 | overhead | 8 |  | 25 |
| Total laboratory | 77 |  | 232 | total laboratory | 77 |  | 232 |
| Feed back to patient | 35 |  | 106 | Feed back to patient | 35 |  | 106 |
| **Total samples 4,6,8** | 250 |  | 749 | Total blood drawing at home | 131 |  | 393 |
|  |  |  |  |  |  |  |  |
|  |  |  |  |  |  |  |  |
|  |  |  |  |  |  |  |  |
| **Sample no. 3, 5, 7 and rest of months [regular samples]** | € | 23 | € | **Sample no. 3, 5, 7 and rest of months [regular samples]** | € | 23 | € |
| Request of the analysis | 9 |  | 204 | Request of the analysis | 9 |  | 204 |
| Laboratory |  |  |  | Laboratory |  |  |  |
| sample | 50 |  | 1150 | sample | 50 |  | 1150 |
| time pharmacist (10 min) | 19 |  | 436 | time pharmacist (10 min) | 19 |  | 436 |
| overhead | 8 |  | 192 | overhead | 8 |  | 192 |
| total laboratory | 77 |  | 1778 | total laboratory | 77 |  | 1778 |
| Feed back to patient | 35 |  | 816 | Feed back to patient | 35 |  | 816 |
| Total samples 3,5,7 and rest | 122 |  | 2797 | Total samples 3,5,7 and rest | 122 |  | 2797 |
|  |  |  |  |  |  |  |  |
| **Total conventional sampling** |  |  | **3972** | **Total DBS home sampling** |  |  | 3553 |

Rumc: Radboud University Medical Center.
Discrepancies between multiplications and or sums may be due to rounding.
