# Supplementary material for: Cost Evaluation of Dried Blood Spot Home Sampling as Compared to Conventional Sampling for Therapeutic Drug Monitoring in Children
Source: PLoS One. 2016 Dec 12;11(12):e0167433. doi: 10.1371/journal.pone.0167433 (PMC5152813; doi:10.1371/journal.pone.0167433)
Supplement: S1 Text — (DOCX) [file pone.0167433.s009.docx]

Supplementary File 1. Literature search, sources of estimates for volume unit and cost units, basic assumptions of the cost analysis.

1. *Literature search*

The following terms were searched in Pubmed, February 24, 2016: ("economics"[Subheading] OR "economics"[All Fields] OR "cost"[All Fields] OR "costs and cost analysis"[MeSH Terms] OR ("costs"[All Fields] AND "cost"[All Fields] AND "analysis"[All Fields]) OR "costs and cost analysis"[All Fields]) AND dried[All Fields] AND ("blood"[Subheading] OR "blood"[All Fields] OR "blood"[MeSH Terms]) AND spot[All Fields]). An Embase search, February 24, on “cost dried blood spot” and on “economic dried blood spot”.

1. *Sources of estimates for volume and unit costs*

The cost estimates for the input parameters were gathered from different sources: literature, interviews with experts, and data from the Radboud university medical center. Data on personnel costs, transport costs and loss of productivity were customary to The Netherlands[1]. Costs were valued from a social, patient, and healthcare perspective.

Sources of estimates for volumes in terms of time spent during each step were gathered from a survey with representatives from patient organizations, pediatricians, nurses and hospital pharmacists (in training). Nurse’s time was estimated based on an interview with two nurses and hospital manager. Pharmacist’ time was estimated based on interviews with hospital pharmacists (n=7). Doctor’s time spend was based on interviews with pediatricians (n= 5). Average waiting time was estimated based on surveys with representatives of patient organizations (n=4). Average travelling distances from home to the hospital per patient reference case were gathered from a consultant from “Consultancy Group Process Improvement and Innovation“ (PVI) of Radboud university medical center and from the guidelines for costs-analysis[1]. The PVI group counsels on processes concerning improvement and changes. The resources of conventional and home sampling for both cases are presented in Tables 1 and 2. All sources on costs and volumes can be found in the supplementary file (Table S1).

1. *Basic assumptions*

The following assumptions were incorporated in the model: (a) All reported costs are in euros (€), 2015 price level. (b) In case only older prices were available, they were recalculated towards 2015 prices using the Dutch consumer price index (CPI) as published by Statistics Netherlands, The Hague/Heerlen[2]. (c) Loss of productivity was valued as loss of paid work for conventional sampling and it was valued as unpaid work (e.g. as informal care) for DBS home sampling. (d) Patients costs are absent in DBS home sampling and equal travel costs in conventional sampling. (e) Costs for the analysis of the samples were set to an integral general cost price of €50. (f) The following costs are not included in the analysis (1) costs on development of the method of analysis in the laboratory (initial capital costs) as they are considered equal for both conventional and DBS sampling; (2) taxes (VAT). (g) On all healthcare costs related to personnel costs, a 44% overhead is calculated, in line with the guidelines for costs-analysis[1].

1. Zorginstituut Nederland. Kostenhandleiding: Methodologie van kostenonderzoek en referentieprijzen voor economische evaluaties in de gezondheidszorg. 2015.

2. Statistics Netherlands, The Hague/Heerlen, Last updated 11-02-2016, Last accessed 02-03-2016, Available from statline.cbs.nl.
